# Pediatric thyroid surgery and management of thyroid nodules – an institutional experience over a 10-year period

**DOI:** 10.1186/s13633-015-0019-x

**Published:** 2016-01-13

**Authors:** Wen Jiang, Robert O. Newbury, Ron S. Newfield

**Affiliations:** Rady Children’s Hospital in San Diego, San Diego, CA USA; Department of Surgery - Division of Otolaryngology, University of California San Diego, 3030 Children’s Way Suite 402, San Diego, CA 92123 USA; Department of Pathology, University of California San Diego, San Diego, CA USA; Department of Pediatrics - Division of Endocrinology, University of California San Diego, San Diego, CA USA

**Keywords:** Pediatric thyroid nodule, Well-differentiated thyroid carcinoma, Thyroid surgery

## Abstract

**Background:**

We reviewed our institutional experience in the diagnosis and management of pediatric thyroid nodules and well-differentiated thyroid carcinoma (WDTC), highlighting the unique challenges in this population.

**Methods:**

IRB approved retrospective chart review was conducted on patients who underwent fine needle aspiration (FNA) or thyroid surgery from 1/1/2001 to 12/31/2010 at Rady Children’s Hospital San Diego, a tertiary referral center in Southern California. Patients thus identified who completed their initial treatment at our institution were included.

**Results:**

Total of 79 subjects qualified; 20 had FNA only, and 59 underwent thyroid surgery. Of the latter, 29 had benign histology and 30 had WDTC. Average age was 14.5 years with a female: male ratio of 4:1 for WDTC versus 1.6:1 for benign nodules. When compared to final pathology, malignancy rate was high in indeterminate FNA cytology at 60 %. Neck metastasis was noted in 40 % and pulmonary spread in 10 % of patients. There was a 2.75 fold increase in malignant cases (*n* = 22) treated during 2006–2010 compared to 2001–2005 (*n* = 8) with more advanced disease at initial presentation. Ninety percent of WDTC patients received adjuvant I-131 treatment with an initial average dose of 132.4 mCi. Despite aggressive initial presentation, 26/30 patients had no evidence of disease at last follow-up, with an average length of 40.3 months.

**Conclusions:**

In recent years, we are managing significantly more WDTC cases at our institution with advanced disease at the onset. Malignancy rate on indeterminate cytology is higher than reported in adults with an overall high rate of malignancy in thyroid nodules removed in this cohort. Disease control and short-term outcome is still excellent. The recently published pediatric guidelines for WDTC will further standardize management.

## Background

Well-differentiated thyroid carcinoma (WDTC) is relatively uncommon in the pediatric population when compared to adults, with those < 20 years old comprising only 1.8 % of all new cases of thyroid malignancy [1]. However, it is not unusual in adolescents and may represent up to 11 % of all new cases of cancer reported in 2014 between the age of 15 and 19 according to the American Cancer Society [2]. Until the recently published pediatric guidelines [3], the management of WDTC in children largely extrapolated from adult guidelines published by the American Thyroid Association (ATA) [4]. However, clinical presentation and tumor behavior may differ significantly in children, with larger volume, earlier involvement of the capsule and more extensive lymph node involvement [5, 6]. The object of this study is to describe the patient characteristics of pediatric thyroid nodules and WDTC presented to our institution and our experience in the diagnosis and management of WDTC over a 10-year period. This case series serves to demonstrate some of the challenges in the pediatric population.

## Methods

### Patient selection

We obtained institutional review board approval for the retrospective review of all patients who underwent thyroid fine needle aspiration (FNA) or thyroid surgery from 1/1/2001 to 12/31/2010 with age ranging from birth to 21 years. A total of 83 patients with thyroid nodules were treated at Rady Children’s Hospital San Diego (RCHSD). One patient with medullary thyroid carcinoma was excluded from the analysis and three patients were excluded due to either previous treatment elsewhere or transfer of care to another institution after the initial biopsy.

### Pre-operative imaging and surgical management

All patients had imaging prior to surgery and all had Ultrasound (US) of the thyroid and neck preoperatively as recommended by the ATA guideline [3]. Seven patients also had Magnetic Resonance Imaging (MRI) for evaluation of regional lymph nodes after the initial US although none of the MRIs revealed any additional information or resulted in change in surgical recommendations. Four patients who presented with bulky neck disease also underwent chest Computed Tomography (CT) without contrast to evaluate for lung metastasis which were all negative. If the thyroid FNA was positive for malignancy, patients underwent total thyroidectomy as the initial surgical procedure. Any patient with clinically palpable cervical lymphadenopathy on physical exam and/or radiologically positive lymph nodes underwent therapeutic comprehensive neck dissection in the involved compartment. Criteria used to define clinically palpable cervical lymphadenopathy included increased size, rounded shape, firmness and poor mobility on physical examination. Suspicious lymph nodes on US had one or more of the following characteristics: increased size, rounded shape, loss of central hilum, cystic appearance, peripheral vascularity on Doppler imaging and micro-calcifications [7]. FNA of cervical lymph nodes were not performed during this study period. Prophylactic central compartment (level VI) neck dissections were not routinely performed at this institution.

### Clinical staging

The sixth edition of the AJCC/UICC TNM classification was used to determine the tumor (T), nodal (N), and distant metastasis (M) stages [8]. Of the 30 patients with malignancy, 27 (90 %) were defined as stage I, and 3 (10 %) were classified as stage II with pulmonary metastasis.

### Follow-up

TSH suppression therapy was administered to all patients with a target of TSH < 0.1mIU/L. Patients were followed using serum thyroglobulin (Tg), neck US and whole body scan (WBS). A patient was considered free of disease at follow-up if basal Tg was non-detectable <0.2 ng/mL as well as stimulated Tg <1 ng/mL (using either T4 withdrawal or Thyrogen), and both US and whole body scan were negative. Prior to 2013, all Tg levels were measured by immunometric assays which maybe inaccurate in the presence of Tg antibodies. Since 2013, for patients with positive Tg antibodies, a new liquid chromatography-tandem mass spectrometry (LC-MS/MS) method [9] (Quest Diagnostics, San Juan Capistrano, CA) has been used to measure serum Tg.

### Statistical analysis

When comparing age differences between FNA-only, benign and malignant group, a one-way Analysis of Variance (ANOVA) was performed and when gender distributions were compared among the three groups, a Pearson’s chi-square test was performed. The differences were considered statistically significant with *p*-value <0.05.

## Results

### Fine needle aspiration

Among the 79 patients who qualified for the study, 20 had FNA-only without subsequent surgery and 59 underwent thyroid surgery. Patient characteristics and tumor sizes are shown in Table 1. Of the 20 FNA-only patients, six samples were initially non-diagnostic, all performed under ultrasound guidance. Of these, two patients had subsequent FNA that showed benign cytology. The other four patients did not undergo subsequent repeat biopsy; two had cystic nodules that resolved after aspiration, and two patients were lost to follow-up after the initial FNA. The cytology of the remaining 14 samples was benign based on the Bethesda classification, including: eight benign nodules, three colloid nodules and three lymphocytic thyroiditis. The average follow-up for this group was 21.4 months with no documented changes in nodule size.

**Table 1:** Patient characteristics

Characteristic	FNA-only	Benign	Malignant	
Number of Pt.	20	29	30	
Age at diagnosis (years)				
Mean (SD) [range]	14.4(3.4) [8–20]	13.2 (3.6) [4–20]	14.6(2.8) [9–21]	*p* = 0.24
Gender				
Male	3	11	6	
Female	17	18	24	
Tumor size, cm				*p* = 0.74
< 2	14	15	10	
2–4	5	12	12	
> 4	1	2	8	
US characteristics				
Cystic components	4 (20 %)	13 (44.8 %)	3 (10 %)	

Among the 59 patients who underwent thyroid surgery, 37 (62.7 %) underwent FNA prior to surgery. Of these, two cases were performed at another institution and were excluded in the FNA analysis. In the remaining 35, the rate of non-diagnostic specimen was 14.3 % (5/35). Indeterminate cytology includes the categories of Atypia of Undetermined Significance (AUS)/Follicular Lesion of Undetermined Significance (FLUS), Follicular Neoplasm and Suspicious for Malignancy. Table 2 shows the finding of FNA when compared to final pathology. Among the FNA samples obtained prior to the publication of the Bethesda staging system in 2008, there was one case of “atypical cytology” that was reassigned as AUS, two cases of “suspicious for malignancy” and two cases of “follicular neoplasm” that remained in the same corresponding categories. Overall malignancy rate of all biopsied nodules including FNA-only patients was 40 % (22/55). In the benign cytology category, three patients were diagnosed with malignancy on final pathology; one patient had a 1.7 x 1.4 x 2.5 complex nodule, one had a 4.7 x 4.2 x 2.7 cm solid nodule and the third patient had 3.8 x 2.8 x 2cm solid nodule.

**Table 2:** Thyroid cytology with implied risk of malignancy: comparing institutional pediatric data to established adult literature

Diagnostic category	% FNA (n)	Number of malignancies	Risk of malignancy RCHSD %	Risk of malignancy Bethesda %
Non-diagnostic	14.3 (5)	1	20	1–4
Benign	17.1 (6)	3	50	0–3
AUS/FLUS	8.6 (3)	1	33	5–15
Follicular neoplasm	20 (7)	4	57	15–30
Suspicious for malignancy	14.3 (5)	4	80	60–75
Malignant	25.7 (9)	9	100	97–99

Final pathology had 62.9 % (22/35) overall malignancy rate out of total of 35 FNAs in the surgical group

*FNA* Fine-needle aspiration, *AUS* Atypia of Undetermined Significance, *FLUS* Follicular Lesion of Undetermined Significance

As a quality control measure, all cytology specimens since 2007 were sent to adult cytologists at University of California, San Diego (UCSD) for review prior to results being released. A total of 24 FNA cytology specimens were sent for second-opinion consultation. Among these, only two samples (8.3 %) had discordant cytological reading. One sample was read as FLUS at RCHSD and benign at UCSD with final pathology being “Hurthle cell adenoma.” Another sample was read as “suspicious for malignancy” at RCHSD and “AUS” at UCSD with final pathology being follicular adenoma.

### Surgery and surgical pathology

Among the 59 patients who underwent thyroid surgery, the group was evenly divided with 29 patients having benign pathology and 30 patients with WDTC. In the benign group, 25 underwent thyroid lobectomy and four patients underwent total thyroidectomy for large multi-nodular goiters with compressive symptomatology. For the patients with WDTC, 19 underwent total thyroidectomy at the initial surgery, and 11 had lobectomy followed by completion thyroidectomy once final pathology confirmed malignancy. Frozen sections were not routinely used for primary tumor given that a large percentage of tumors with indeterminate cytology were follicular neoplasms where frozen sections are not helpful determining vascular or capsular invasion. Among the WDTC, the majority 18/30 (60 %) were classic papillary carcinoma, 8/30 (26.7 %) were follicular variant of papillary carcinoma and 4/30 (13.3 %) were follicular carcinoma.

The mean age at presentation was 13.9 ± 3.7 years in all patients who underwent thyroid surgery, with a range of 4 to 21. There was no statistical difference in the mean age between the benign and malignant groups (13.2 and 14.6 years respectively) (Table 1). When gender differences were analyzed, there was a female predominance in the WDTC group with a female to male ratio of 4:1 (24:6) when compared to the benign pathology group 1.6:1 (18:11). However this was not statistically significant. With respect to ethnicity, in our study population, 63 % of the patients were Hispanics by self-reporting with no significant differences between benign (52 %) and malignant (73 %) groups. Patients were from over 26 zip code areas throughout San Diego County and neighboring counties, and we did not notice any obvious geographic clustering.

**Fig. 1:**
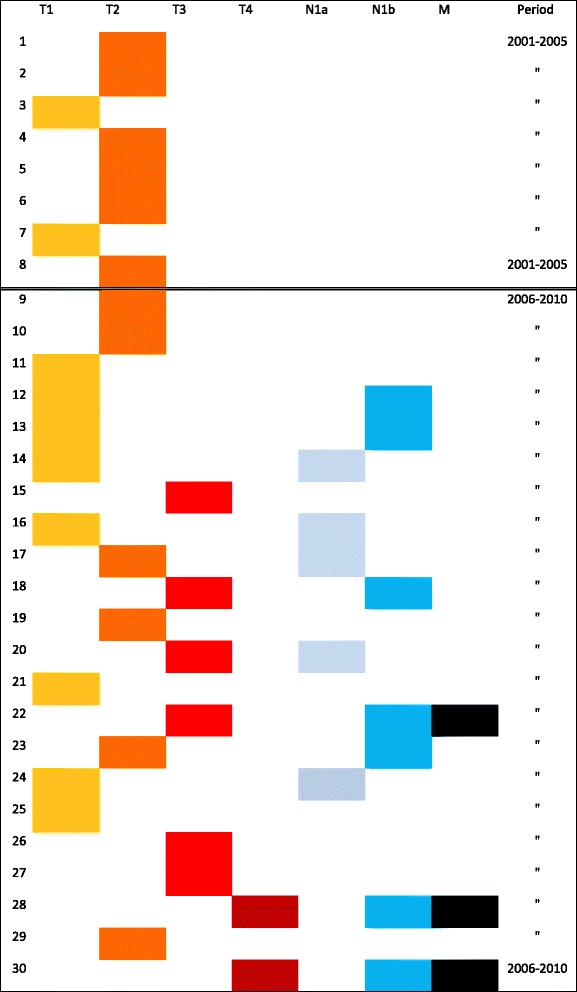
TNM Staging of WDTC in 30 consecutive patients from 2001 to 2010. The sixth edition of the AJCC/UICC TNM classification was used to determine the tumor (T) stage, nodal (N) stage, and distant metastasis (M) stage

Overall, 40 % of the patients had regional metastasis determined by positive nodal disease on final pathology and 10 % had pulmonary metastasis as evident by diffuse uptake in the lung on whole body scan (WBS) after the initial radioiodine (I-131) treatment. Of the 30 patients with malignancy, 27 (90 %) were defined as stage I, and 3 (10 %) were classified as stage II with pulmonary metastasis. Figure 1 illustrates the TNM Staging of patients with WDTC. All three patients with pulmonary metastasis had large primary tumors and bulky lateral neck N1b disease consistent with the pattern of metastatic spread for papillary thyroid carcinoma [10]. Forty percent (12/30) of the patients underwent central and/or lateral compartment neck dissection. Seven out of 30 patients underwent both central neck dissection and selective lateral compartment neck dissection most commonly involving level II-IV. Five additional patients underwent central neck dissections only. All seven patients who had N1b disease presented with bulky palpable neck mass at the initial clinical exam ranging from 1.5 to 4 cm that was confirmed with both US and MRI prior to undergoing comprehensive lateral compartment selective neck dissections. These were felt to be obvious metastatic disease; therefore, FNA was not performed pre-operatively. There was also bulky central neck disease in these seven patients, consistent with the pattern of spread for papillary thyroid carcinoma. In the absence of lateral neck disease, central neck dissections were performed based on the surgeon’s determination of pathologic nodal disease intra-operatively with judicious use of frozen sections. Prophylactic central compartment (level VI) or lateral neck dissections were not routinely performed at this institution. One patient was included in node positive staging group with a microscopic focus of papillary carcinoma in a lymph node incidentally removed with total thyroidectomy specimen without formal neck dissection. When neck dissections were performed, only one case was negative for nodal metastasis in the central compartment due to the discrepancy between the pre-operative FNA of papillary thyroid carcinoma and the final pathology of follicular carcinoma, which rarely metastasize to regional lymph nodes.

### Postoperative complications

With regards to surgical complications, two of the 59 patients who underwent thyroidectomy had unilateral vocal cord paralysis (3.4 %). One was anticipated due to gross extra-capsular spread with direct tumor involvement (T4) of the recurrent laryngeal nerve at the time of surgery. The same patient also suffered permanent hypoparathyroidism as a consequence of extensive central neck involvement, the only patient in the study population to develop that complication (1.7 %).

### Radioactive iodine and follow-up

Post-operatively, the majority of the patients with WDTC (90 %) were treated with radioactive iodine (I-131), with an average initial dose of 132.4 mCi. Prior to 2008 and the capability to administer I-131 on an outpatient basis at RCHSD, all patients were referred to outside institutions for radioiodine therapy. Three patients received additional I-131 due to persistently elevated Tg from pulmonary disease after the initial treatment. One patient with T4N1b disease required re-operation due to persistent disease 6 months after the initial total thyroidectomy and ipsilateral central and lateral neck dissections. She had persistently elevated Tg 3 months after 169 mCi of I-131 treatment and US revealed a para-tracheal mass and contralateral suspicious central compartment lymph nodes. She had re-operation in the thyroid bed and contralateral central neck dissection at the same time that was not addressed at the initial surgery.

The average follow-up was 40.3 ± 24.9 months for patients with WDTC with a range of 4 to 98 months. In the entire cohort, there was no regional nodal recurrence in the operated compartment at the last follow-up. Despite aggressive presentation, 26/30 patients had no evidence of disease by non-detectable basal and stimulated Tg, US and/or WBS at the time of the last follow-up.

## Discussion

WDTC is relatively uncommon in the pediatric population; however the overall incidence is rising in recent years. The reported incidence was 0.54 per 100,000 in 2004 according to the Surveillance, Epidemiology and End Results (SEER) database [5] with more recent data showing the average age–adjusted annual incidence (2008 to 2011) has risen to 0.89 per 100,000 persons in < 20 years old group. In this series, the mean age of presentation is 14.6 for WDTC which is slightly younger than the peak incidence between 15 to 19 reported in the adolescent age group [5]. The female predominance (4:1) found in our study population is in agreement with the overall national incidence of thyroid carcinoma with age-adjusted incidence of 0.89 cases per 100,000 in females and 0.20 cases per 100,000 in males [1]. Among the patients with WDTC, the most common histologic type is classic papillary carcinoma 18/30 (60 %), followed by follicular variant of papillary carcinoma 8/30 (26.7 %) and follicular carcinoma 4/30 (13.3 %). This is also consistent with published literature with majority of pediatric WDTC being papillary pathology [10].

Our institution is located in Southern California north of the border between the United States and Mexico with 45 % Hispanic children in San Diego County according to census data from 2008 to 2012 [11]. In our study population this proportion is even higher at 63 %. The high percentage of Hispanics makes our study population somewhat unique and may affect the generalizability of the study result in other geographic areas with different demographics.

In the FNA-only group, the non-diagnostic rate is high at 30 % compared to the surgical group of 14.3 %. The follow-up of these patients was not always adequate, due to age and relocation. In order to address the high non-diagnostic rate, we have instituted mandatory adequacy testing at the time of the FNA procedure in 2012 and have reduced our overall non-diagnostic rate to 0 % for the last 2 years.

In the surgical group, FNA was performed on the primary thyroid nodule in majority (62.7 %) of the patients prior to surgery. With the recent availability of Tg assay from FNA washout at our hospital, we are starting to incorporate the recommendation of obtaining cytological confirmation of metastatic disease to lymph nodes in the lateral neck prior to surgery according to the 2015 pediatric guidelines [3]. Most of the patients who were not offered FNA prior to surgery were from earlier years when the surgical staffs at our institution performed primary excisional biopsy or lobectomy for tissue diagnosis due to concern for malignancy and accuracy of pediatric FNA. Prior to 2007, thyroid surgeries were performed by surgeons from various disciplines including pediatric surgery, pediatric otolaryngology and adult endocrine surgery who came to RCHSD occasionally, and each surgeon only performed 1–2 procedures a year at our institution. Since 2007, the evaluation at our institution has been streamlined and standardized. A single surgeon performed 90 % of all thyroid surgeries, and all patients with thyroid nodules were offered FNA when indicated prior to surgery. A formal multi-disciplinary pediatric thyroid tumor board was established during the later part of the study period where imaging, FNA results, surgical planning as well as adjuvant therapies were formally discussed for every patient following the existing ATA guidelines [4]. We realized that due to the limited volume of thyroid FNAs performed at pediatric institutions, our cytologists might not have the vast experience of their adult colleagues. As a quality control measure, currently all cytology specimens are sent to adult cytologists at UCSD for review prior to results being released. The concordance between the second opinion at UCSD and the initial FNA reading at RCHSD reading is excellent at 91.7 %.

At an initial glance, our institutional rate of malignancy is quite high in the benign and indeterminate categories when compared to published data [12]. The published data in the Bethesda staging system is based on the adult population with malignancy rate of 5–15 % [13], whereas in our population more than half of the nodules removed surgically were malignant. A recent publication from Boston Children’s hospital reported a cancer rate of 22 % in their population [14]. However, majority of their patients (63 %) had benign cytology on FNA and did not undergo surgery; therefore the false negative rate of the cytology could not be assessed. In their indeterminate cytology categories, the cancer rate is similar to our finding ranging from 40 to 100 % [14] when compared to final pathology. Despite this discrepancy between the pediatric and adult population, we still believe that FNA should always be performed prior to surgery, as recommended in the recently published pediatric guidelines [3]. Given our excellent institutional accuracy for “malignant” and “suspicious for malignancy” cytology categories, total thyroidectomy is offered up front for these patients eliminating the need for a second completion surgery. Two of the three cases of the false negative FNAs involved large nodules where the accuracy of FNA may decrease [15]. Based on our experience and published studies [14], when FNA result is either benign or indeterminate for nodules ≥ 4 cm, at a minimum thyroid lobectomy should be strongly considered, and it is perhaps not unreasonable to consider total thyroidectomy after careful discussion with the family presenting pros and cons of each option. Among the lesions with AUS/FLUS and follicular neoplasm on FNA, significant numbers (4/10) were follicular variant of papillary thyroid carcinoma, a diagnosis that is known to present a challenge with FNA [16].

We observed a significant increase in the number of malignant cases treated at our institution during the second half of the study period compared to the first half. For benign nodules, there was a small increase from *n* = 12 (2001–2005) to *n* = 17 (2006–2010). In comparison, there was a 2.75-fold increase in malignant nodules during that time, from *n* = 8 (2001–2005) to *n* = 22 (2006–2010). During the same period, the incidence of thyroid cancer in the San Diego region increased 1.68-fold from 7.13/100,000 to 12.31/100,000 according to the California cancer registry [17] which mirrors the increase in national incidence according to the SEER database [1]. The more dramatic increase at our institution may be explained, in part, by a shift in referral pattern. We speculate that pediatric patients that previously had been sent to adult centers with higher thyroid surgical volumes were referred to RCHSD during the latter portion of the study period. By establishing a thyroid tumor board where pediatric specialists across disciplines meet and discuss management, we were able to provide streamlined and standardized care where guidelines and best practices are closely followed. We believe that by building a “center of excellence,” we have attracted more referrals of higher complexity cases from the community. Most of the national increase in adults in recent years is generally attributed to early detection of small T1 nodules due to increased US sensitivity [18, 19]. However this is not the case with our population. Figure 1 shows the pathologic staging of all 30 consecutive cases during the study period between 2001 and 2010. There is an increase in the number of cases managed at our institution, and also more aggressive presentation with T3/T4 lesions, positive nodal and pulmonary metastasis. In the second half of the study, there were a total of six T3 and two T4 tumors that were not seen during the first half. All seven patients with N1b disease presented with bulky neck mass at the initial visit. Among the patients with positive N1a disease, four patients did have relatively small primary tumors, three T1 and one T2 nodules. For these four cases, the performance of the central neck dissection may have up-staged the patients compared to earlier years where comprehensive neck dissections were not performed. These cases may have contributed to the increased overall staging of the cohort due to surgeon practice or detection bias.

With regards to surgical complications, we are unable to report the rate of transient recurrent nerve paralysis or hypocalcemia in the postoperative period due to lack of systematic documentation during this long study period. However, since 2007, all thyroidectomy patients at our institution are required to have both pre-op and postop flexible laryngoscopy exam with clear documentation of vocal cord mobility. Protocols are in place now to document systematically both serum calcium and PTH level postop and at subsequent outpatient follow-ups. Future analyses will be able to report on these points as a measure of surgical outcomes.

## Conclusions

There has been a substantial increase in the number of WDTC cases referred to our institution in recent years with more advanced TNM staging at the time of initial presentation. There is a strong female predominance in a largely Hispanic population. Despite the advanced disease in some cases, both local regional control and short-term outcomes are excellent with comprehensive thyroidectomy surgery, appropriate therapeutic neck dissections followed by adjuvant I-131 and suppressive levothyroxine therapy.

Finally, long-term follow-up is essential to evaluate ultimate prognosis in the treatment of WDTC in pediatric population with disease specific survival and recurrence rate measured in decades instead of years. Since most of our patients present in adolescence, their care often transitions to adult institutions several years after completion of their treatment making follow-up data difficult to obtain, not to mention patient relocation. This is a challenge faced not only by pediatric specialists, but also by many adult centers. In order to follow these patients longitudinally, a national database needs to be established where a patient’s records can be carried over institutions. Secure data sharing and health information exchange through Electronic Health Records technology will also be helpful.

We have highlighted some of the challenges in the management of thyroid nodules and treatment of WDTC in the pediatric population. The newly published pediatric guideline for WDTC by the ATA should help standardize future management and treatment [3].
